# Multi-omics integration strategies for animal epigenetic studies — A review

**DOI:** 10.5713/ab.21.0042

**Published:** 2021-04-23

**Authors:** Do-Young Kim, Jun-Mo Kim

**Affiliations:** 1 Department of Animal Science and Technology, Chung-Ang University, Anseong, Gyeonggi 17546, Korea

**Keywords:** Epigenetic, Methylome, Transcriptome, Multi-omics Integration Analysis, Gene Regulation

## Abstract

Genome-wide studies provide considerable insights into the genetic background of animals; however, the inheritance of several heritable factors cannot be elucidated. Epigenetics explains these heritabilities, including those of genes influenced by environmental factors. Knowledge of the mechanisms underlying epigenetics enables understanding the processes of gene regulation through interactions with the environment. Recently developed next-generation sequencing (NGS) technologies help understand the interactional changes in epigenetic mechanisms. There are large sets of NGS data available; however, the integrative data analysis approaches still have limitations with regard to reliably interpreting the epigenetic changes. This review focuses on the epigenetic mechanisms and profiling methods and multi-omics integration methods that can provide comprehensive biological insights in animal genetic studies.

## INTRODUCTION

Genome-wide studies have provided considerable insights into the genetic basis of inheritance; however, they could only partially explain the heritability of complex traits [1]. Complex traits in livestock can be attributed to genetic factors; however, the final phenotypic outcome is highly dependent on the farm environment, ecotypes, and individual genetic backgrounds. This missing heritability in complex traits can be attributed to the contribution of epigenetic variability, arising from the interactions with environmental factors. Transcription, translation, and the subsequent protein modification constitute the transfer of genetic information from an archived copy of DNA to mRNA with subsequent protein production. Every cell in an organism essentially has the same DNA sequences, but the qualitative and quantitative differences in gene expression determine the cell types and functions. Regulation of gene expression is the key to differentiation and development. Epigenetics can be defined as the inheritable changes that modify DNA or related proteins without altering the DNA sequence [2]. Epigenetic information is encoded in the gene sequence and is regulated through DNA methylation, histone modification, and RNA interference (RNAi) [3]. Various epigenetic mechanisms regulate gene expression by modulating the access of transcription factors (TFs) to the regulatory regions of the gene.

DNA methylation, histone modifications, and RNAi regulate gene expression through interactions with the genetic and environmental factors, in turn influencing the phenotype, resulting in variations in various biological mechanisms. Identifying and understanding the mechanisms of epigenetics is important in a variety of traits, such as disease and productivity. Epigenomics provides new insights in cell biology. The development of microarray and NGS technologies enable generating genome-wide epigenetic data from large populations for investigating the characteristics of organisms and their interactions with the environment.

Epigenetic research has the potential to unravel the mechanisms underlying gene regulation through interactions with the environment; however, it remains underutilized. The epigenetic profile is continuous, dynamic, and spatiotemporally tissue-dependent, similar to the transcriptome. The advancement of NGS technology enables generating large amounts of epigenetic data and developing data analysis approaches for identifying and interpreting epigenetic changes. This review aims to discuss the key mechanisms of epigenetic regulation and the various omics data analysis methods for the characterization of the epigenetic factors throughout the genome.

## EPIGENETIC MECHANISMS

**Figure 1:**
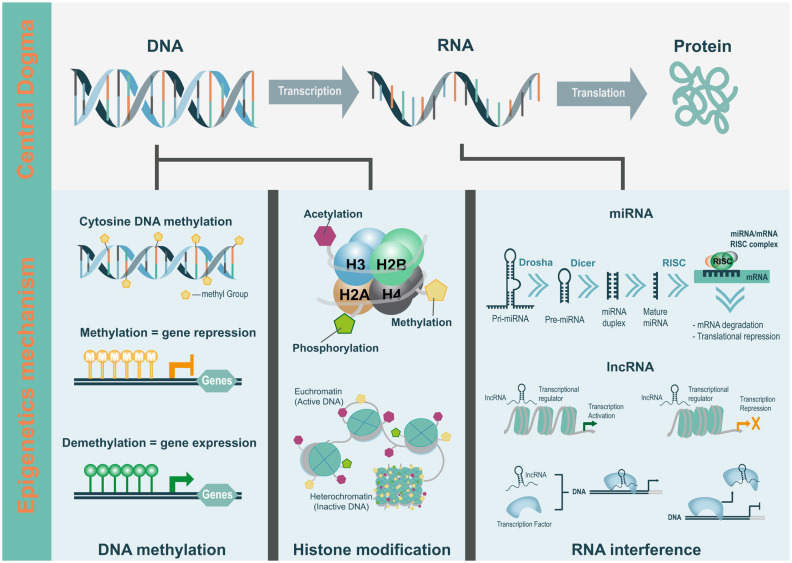
Genetic regulation overview by epigenetic mechanisms according to the central dogma.

The functional unit of gene expression is the chromatin; it is made up of basic units called the nucleosome, which is a complex of DNA and histone proteins. Modifications of DNA and histone proteins alter chromatin structure and subsequently influence gene expression. It is important to understand the process of reversible changes in gene activity, which are modulated through various epigenetic mechanisms (Figure 1).

### Histone modification

Histone is the core protein component of the chromatin complex; it provides a structural backbone for the DNA to wrap around at regular intervals to form the chromatin. The nucleosome represents the first level of chromatin organization. It is composed of two copies each of histones H2A, H2B, H3, and H4, assembled in an octameric core with DNA tightly wrapped around the octamer [4]. Nucleosomes are unstable and change rapidly in response to external stimuli, often leading to permanent changes and contributing to disease development and progression. Modifications such as acetylation, methylation, ubiquitylation, phosphorylation, sumoylation, ribosylation, and citrullination occur post-translationally in the amino acids of the histone proteins; acetylation and methylation are the most extensively studied histone modifications [5,6].

Histone acetylation occurs in the amino group of specific lysine residues at the N-terminus of histone proteins [7]. Histone acetyltransferases (HATs) add acetyl groups to the tail lysine residues of histones; on the other hand, histone deacetylases remove acetyl groups from an acetylated lysine [8,9]. The role of histone acetylation in transcription was evaluated by identifying the causal relationship between histone acetylation and gene transcription. Several transcription cofactors have unique HATs [7,9]. HATs focus on specific gene promoters through interactions with DNA-binding regulatory factors, resulting in targeted acetylation and activation of transcription [10]. There are two mechanisms of histone acetylation, namely, charge neutralization and protein recognition/recruitment related to transcription activation. The charge neutralization method neutralizes the positive charge in the lysine side chain, disrupting the interaction between the negatively charged DNA backbone and the lysine residue. As a result, the chromatin gets compressed, and the efficient binding of the TF to the transcription initiation site is affected [11]. The protein recognition/recruitment method causes certain histone tail acetylation patterns and other modifications in distinct sets of regulating proteins to regulate chromatin structures and functions [12–14].

Histone methylation involves transferring methyl (−CH_3_) groups derived from S-adenosyl methionine to the amino acids lysine and arginine. Histone methylation is catalyzed by histone methyltransferase (HMT) and the demethylation by histone demethylase. Lysine can be mono-, di- or tri-methylated; arginine can be mono-, or symmetrically or asymmetrically di-methylated [15–17]. Methylation and demethylation of histones result in the activation or inhibition of gene expression, respectively, by modulating the access of DNA to the TFs, through loosening or wrapping of the histone tail [18]. Histone methylation is predicted to be stabler than other modifications under physiological conditions; and therefore, this stability increases the possibility of histone methylation being permanent. Many histone methylations are reported in mature chromatins [11].

In contrast to histone acetylation, histone methylation does not affect the charge of histone proteins. Histone acetylation is generally correlated with transcription activation; however, histone methylation modulates transcription activation or inhibition, depending on the specific amino acid on the histone protein that is modified. Different parts of chromatin can be activated or deactivated by histone modification, depending on the methylation site [19,20]. Among the various histone methylation regions, methylation in the H3-K4 and H3-K9 regions is the most widely studied. Di- and tri-methylation of histone H3 at lysine 4 (H3-K4) region is associated with transcriptional activation, similar to the acetylation of histone H3 at lysine 14 (H3-K14) region [21, 22]. However, di- and tri-methylation of histone H3 at lysine 9 (H3-K9) region results in chromatin condensation and subsequent transcription inhibition [23,24]. This region is the target for opposing outcomes; and therefore, the two modifications are mutually exclusive in their positioning within the chromatin.

### RNA interference

The RNAi is the most recently discovered mechanism affecting epigenetic changes. Cell differentiation is modulated by regulating the expression at the gene level and chromosome level through non-coding RNAs (ncRNAs) [25–29].

The ncRNAs are not translated into proteins and are classified into housekeeping ncRNAs and regulatory ncRNAs. Regulatory RNAs are classified, based on their size, into short non-coding RNAs (sncRNAs), such as siRNAs, miRNAs, and piRNAs, and long non-coding RNAs (lncRNAs). In this study, we have reviewed miRNAs and lncRNAs, among the various ncRNAs, because they have been studied extensively for their roles in the regulation of gene expression [30,31].

miRNA is an evolutionarily conserved small single-stranded molecule (approximately 24 nucleotides). It is present in approximately 50% of the chromosomal regions prone to structural changes at the post-transcriptional level [32]. miRNAs regulate hundreds of different genes [33–36]. Unlike other small RNAs, miRNAs are derived from transcripts that form a unique hairpin structure [37]. pre-miRNAs, forming the hairpin structure, become mature miRNAs and form RNA-induced silencing complexes [38,39]. The miRNA base pairs with the mRNA through complementarity, resulting in translation inhibition or deadenylation and degradation in the 3′-untranslated region [38,40]. A study on 13,000 human genes speculated that the potential targets of miRNAs are HMTs, methyl cytosine phosphate guanine (CpG)-binding proteins, chromatin domain proteins, and histone deacetylases [34].

lncRNAs are 200 nt or more in length and include most non-protein-coding transcripts [41]. lncRNAs are used according to the proximity to the protein-coding genes: i) sense or ii) antisense, when there is an overlap of one or more exons of another transcript on the same or opposite strand, respectively, iii) bidirectional, when the expression of the target gene and that of a neighboring coding transcript on the opposite strand are initiated in close genomic proximity, iv) intronic, when it is derived entirely from within an intron of a secondary transcript, or v) intergenic, when it lies within the genomic interval between two genes. In addition, lncRNAs have various origins, such as: i) arising from the disruption of translational reading frame of a protein-encoding gene; ii) resulting from chromosomal reorganization; for example, by the joining of two non-transcribed DNA regions in a manner that promotes transcription of the merged, non-coding sequences; iii) produced by replication of a non-coding gene by retrotransposition; iv) generation of a ncRNA containing adjacent repeats through partial tandem duplication; and v) arising from the insertion of transposable element(s) into a gene in a way that produces a functional, transcribed ncRNA [41]. There are no common shared mechanism in the lncRNA occurrences; however, they play similar roles in the regulation of gene expression [42]. Some lncRNAs may represent transcriptional noise or experimental artifacts; on the other hand, others serve as precursors of short RNAs; however, in many cases, they appear functional in the actual transcripts, mostly auto-regulating their own expression. Evidence that many lncRNAs are functional is confirmed through evolutionary choices regarding tissue specificity, regulation during development, localization to specific cell compartments, and association with diseases [43].

### DNA methylation

DNA methylation is an epigenetic mechanism extensively studied in plants and animals. DNA methylation is the covalent modification at the C-5 position of a cytosine residue in the DNA strand, resulting in 5-methylcytosine (5mC) [44,45]. In mammalian somatic cells, 98% of DNA methylation occurs in a CpG sequence; on the other hand, in embryonic stem cells, only 75% of DNA methylation occurs in CpG [44]. In addition, a significant proportion of DNA methylation is detected in non-CG sites (CHG or CHH; where H can be A, T, or C), other than CpG [46,47]. These differentially influence the gene structure and function [48]. In mammals, transcription of most protein-coding genes is initiated at a promoter rich in CpG sequences. These CpG sequences when present in high density are known as CpG islands (CGI). There are approximately 29 million CpGs in the human genome, of which 60% to 80% are methylated [34,46]. Approximately 70% of annotated gene promoters are CGI-related, and CGI is mostly resistant to DNA methylation [49]. The chromatin structure adjacent to the CGI promoter facilitates transcription; on the other hand, methylated CGI causes chromatin condensation, inhibiting the onset of transcription and subsequently the gene expression. Methylation in genes is positively correlated with gene expression and may stimulate elongation and splicing [50]. In addition, DNA methylation plays a key role in normal development, genomic imprinting, X-chromosome inactivation, chromosome stability, and suppression of repetitive element transcription [44,51]. DNA methylation is regulated by DNA methyltransferases (DNMTs), such as DNMT1, DNMT3A, DNMT3B, and DNMT3C [50].

DNA methylation is chemically and genetically stable; however, it is a reversible modification that can occur either actively or passively. Demethylation involves the oxidation of ten-eleven translocation family enzymes (TETs) from 5mC to 5-hydroxymethylcytosine (5hmC), which is further oxidized to 5-formylcytosine (5fC) and 5-carboxlcytosine (5caC) [52]. The genome-wide distribution of 5hmC differs from that of 5mC. For example, in the adult human brain, 5mC is present in most of the gene regions; on the other hand, 5hmC occurs mainly in the promoter regions [53], regulating gene transcription or translation. A larger number of 5hmC molecules are associated with the bodies of active genes and they are often observed at the transcription start site of genes with a promoter containing a high CpG content. The 5hmC and TET proteins may modulate gene expression by regulating the chromatin accessibility of the transcriptional machinery or by inhibiting repressor binding. This is consistent with the enrichment of 5hmC in the gene body, promoter, and TF-binding regions [54]. DNA methylation can, therefore, serve as a distinct epigenetic marker owing to its functional role in transcriptional regulation.

## TRANSCRIPTOMIC AND EPIGENOMIC PROFILING

Pyrosequencing, polymerase chain reaction (PCR), and Sanger sequencing are widely used to analyze a transcript and its methylation. These methods are precise and useful; however, they can be used only in a specific region, for identifying a small number of gene expressions. This is a limitation for evaluating a large number of samples, because of the high running time. With technological development, powerful approaches have emerged to compensate for these shortcomings.

### Transcriptome profiling methods

Methods such as microarray and RNA-Seq provide a comprehensive understanding of whole-genome transcripts. These methods generate large amounts of expression data that require biological interpretation. In addition, each method has unique characteristics and requires a different analysis. Table 1 summarizes the differences between microarray and RNA-Seq techniques [55,56].

Microarray technique was developed to monitor the expression of multiple genes simultaneously. The technique originated from the large-scale mapping of genomic DNA and sequencing. Microarrays can be classified into printed, *in-situ* synthesized, high-density bead, or electronic, based on the characteristics of the oligo or probe, target detection, and surface support [57]. The basic principle of a microarray involves hybridization between complementary DNA strands when DNA strands (short oligos) or probes of the gene (or region to be detected) are arranged on a microchip and a fluorescently labeled target transcript is added. The transcript abundance of a specific gene or RNA is determined based on the fluorescence intensity of each probe or short oligo, and its location on the chip provides information about the target. Microarrays generate quantitative data that yield information about the qualitative data. However, the microarray technique can only identify genes that are previously reported; it cannot predict novel or un-identified genes, and the normalization process is affected by technological variations, rather than biological differences [58].

RNA-Seq profiles the whole transcriptome, and it is the most suitable method for evaluating the expression of transcripts. Compared to the microarray methods, RNA-Seq has less background noise and wide dynamic range, and enables the detection of quantitative expression, rather than relative values. It is not limited to genomic sequences; and therefore, enables the discovery of previously unknown genes and new isoforms [59]. In addition to standard RNA sequencing methods, various types of transcriptome profiling methods exist, such as DGE-seq [60], useful for profiling specific gene expression; targeted RNA-Seq, for the detection of under-expressed genes [61]; single-cell RNA-Seq, for transcriptome studies at the single-cell level [62]; and micro RNA-Seq, for the detection of small ncRNA (less than 30 bp) [63]. Various platforms can be used for the analysis of RNA-Seq data, depending on the purpose and the method.

RNA-Seq involves preparing an RNA sample, synthesizing its cDNA, fragmenting the RNA, and attaching sequences essential for sequencing (such as adapters) to both ends of the fragment, for generating a library. The template strands are amplified to form clusters, and emulsion PCR methods or enzymatic amplification methods are used, depending on the platform. The sequence of the amplified template strand is analyzed using the NGS technique, and the biological significance of the generated data is evaluated for understanding the molecular mechanisms at the transcript level.

### DNA methylation profiling

Changes in DNA methylation patterns are well-known mechanisms of epigenetic modification. The development of NGS technology and sequencing-based DNA methylation profiling methods enable mapping complete DNA methylomes. Three methods are available for detecting the DNA methylation in the genome and generating methylation data. They include restriction enzyme-based approaches such as *Hpa*II tiny fragments Enrichment by Ligation-mediated PCR (HELP) [64], and methylation-sensitive restriction enzyme (MRE)-seq [65]; bisulfite conversion-based approaches such as whole-genome bisulfite sequencing (WGBS) [46,66], reduced-representation bisulfite sequencing (RRBS) [67], and bisulfite sequencing (BS-seq) [68]; and affinity-enrichment based approaches such as methylated DNA immunoprecipitation and sequencing (MeDIP-seq) [65,69] and methylated-CpG-binding protein sequencing (MBD-seq) [70]. These provide consistent results; however, the most appropriate approach should be chosen, based on the specific biological points to be addressed. This is because the extent of genomic CpG coverage, resolution, quantitative accuracy, and cost vary widely [71–74], among the various methods. Detailed characteristics of the most commonly used genome-wide approaches are described in Table 2.

Restriction enzyme-based method uses a reagent that selectively binds to methylated DNA or cleaves DNA when it is not methylated. The MREs such as *Msp*I, *Hpa*II, *Not*I, and *Sma*I, form the basis of restriction enzyme-based methods.

The HELP assay restricts genomic DNA using a MRE, but employs a methylation-insensitive isoschizomer *Msp*I as a control [75]. It provides better accuracy for both microarray and NGS-based analyses [76–78]. The control *Msp*l expression is affected to the same extent, regardless of methylation status, fragment size, or mutations in the locus; and therefore, expressing the *Hpa*II signal in each gene sequence enables a better comparison among the different gene sequences in the same DNA sample [75].

In MRE-seq, only 40 to 220 bp DNA fragments can be sequencing, and the methylation status is confirmed based on the restriction of the unmethylated region. This is a time- and cost-effective sequencing method; however, it has the limitation of not reconciling the region of interest, because of the reliance on only a limited area of the genome [79].

The most common way to distinguish between methylated and unmethylated cytosine is to convert the unmethylated cytosine to uracil by treating the DNA with sodium bisulfite, while preserving the methylated cytosine [66]. Following the conversion, the uracil is converted to thymine in the PCR step. The WGBS is the most informative and accurate method that covers the entire genome theoretically and is often used to investigate the regions outside of CGI [71,79]. It is also the most direct method with the highest resolution, for detecting methylation across the entire genome. However, it is the most expensive and a resource-demanding technique; therefore, this highly efficient method can only be employed when a comprehensive DNA methylation profile is required [80].

The RRBS is used to reduce the experimental cost of WGBS. RRBS is effective in identifying methylation of specific regions where CpG loci is dense, rather than that of the entire genome. The RRBS method is similar to WGBS; however, CpG-rich fragments are selected prior to bisulfite conversion and the PCR of unmethylated cytosines. Selection of fragments that are 40 to 220 bp in length covers 85% of the CGI in the promoter region [79].

Affinity-based capture methods are proposed as a cost-effective alternative for sequencing only the methylated portion of the genome. In this approach, genomic DNA is fragmented and the methylated fragments are bound to either antibodies [81] or proteins with a high affinity for methylated DNA [70]. Subsequently, the unmethylated fragments are removed, and the methylation-rich portion of the genome is selected and sequenced. Depending on the affinity-based capture method used, the analytical properties may vary depending on the DNA-binding protein or antibody used.

The MeDIP-seq method employs anti-methyl cytosine antibodies. Briefly, genomic DNA is sonicated, adapters ligated to the fragments, samples denatured, and the immunoprecipitated fragments analyzed using antibodies against methylated cytosine. The immunoprecipitated DNA represent the methylated portion of the genome and is identified by comparing with the reference genome [65].

The MBD-seq is similar to MeDIP-seq, with the exception that it does not involve denaturation [73]. In addition, unlike the MeDIP method, which captures DNA fragments containing methylated cytosine, the MBD-seq method uses a protein that binds strictly to methylated CpG. MBD-seq is comprehensive with only a few exceptions, because DNA methylation in most mammalian body tissues occurs almost exclusively in CpG dinucleotides. MBD-seq is more effective in identifying methylated regions containing multiple methylated cytosines; on the other hand, MeDIP-seq is effective in identifying regions with sporadic methylated CpG with low biological relevance [70].

## MULTI-OMICS INTEGRATION ANALYSIS

To understand the molecular complexity that underlies the various phenotypes, it is important to understand each molecule's interactions and the changes at the different molecular levels, such as at the genome, transcriptome, proteome, and metabolome levels. With the advancement of NGS technology, many biological data sets are produced at a rapid rate; however, data analysis, for providing biological insights, remains a challenge. Multi-omics integration (MOI) approach could provide comprehensive and extended biological insights. In studies using various omics data, the approach for MOI is largely focused on the statistic- and the function-based integration methods. This section describes epigenetic studies using MOI. We introduce the MOI analysis method using omics data at different molecular levels that can be applied to epigenetic research (Table 3).

### Epigenetics analysis using MOI strategy

Epigenetic studies based on DNA methylation data are widely employed in humans, livestock, and plant species. Most epigenetic studies use single data on the mechanisms of DNA methylation, histone modification, and RNA interference. Most of these studies only provide predictions of the impact on gene expression, based on the changes in epigenetic mechanisms. High-resolution and high-throughput data are generated with advances in technology; however, there is difficulty in establishing an effective approach that can facilitate combined data analysis with other omics data. Here is a summary of the recent observations of epigenetic changes in multi-omics data using a variety of data integration analyses.

Transcriptome and methylome data are generally used for evaluating epigenetic changes using the MOI method. A common method of integration is to use gene overlap of multiple omics data. There are three ways of gene overlap: i) Entire overlap between the identified differentially methylated genes (DMGs) and differentially expressed genes (DEGs), ii) overlap based on methylation and expression levels, iii) overlap between DMGs and DEGs based on gene components [82–84]. The entire overlapping method is a simple and intuitive method for finding the relationship by using the overlapping genes of the entire DMGs and DEGs. The overlapping method based on methylation and expression levels checks positive and negative associations through the overlapping genes of each level divided into DMGs (hypo-, hyper-) and DEGs (up-, down-). Overlapping based on gene component distinguishes DMGs according to each gene component, and the association between the gene component methylation and gene expression is confirmed through the overlapping. However, the accuracy is compromised based on the method of producing genetic data and the DMG profiling tool used; in addition, this technique is difficult to use when there are no overlapping genes.

In addition to the gene superposition methods, various databases and methods are used for direct (physical) and indirect (functional) analyses [85–87]. The most common is to build a PPI network, a gene interaction network that identifies sets of genes that can interact with each other, using the STRING database [83,88]. Using the TF database, we can construct networks that provide the orientation and relationship to the target gene along with the motif enrichment analysis [89]. Finally, the network can be configured using statistical correlation coefficients. Constructing a network helps understand the direct relationship between the two genes and the mechanisms by which they are indirectly regulated. These networking methods form sub-clusters so that genes that are critical for the function and mechanism can be identified. In addition, for some genes, they provide a broader understanding of epigenetic changes, allowing exploration on directionality and potential regulatory relationships. The limitation is that this networking method is often configured using a reference database; and therefore, it may not be suitable for lesser-studied species.

### Statistic-based integration

The statistic-based integration method is classified into three subgroups: i) correlation-based, ii) clustering-based using data set connection, and iii) multivariate analysis. The correlation-based integration approach finds the relationships between elements in one data set and those in another. The advantage of this integration is its simplicity and intuitiveness. Correlation-based integration is mainly performed using Pearson and Spearman correlation coefficients, which evaluate linear and ranking relationships; correlation analysis can also be performed using other methods, which provide standard correlation coefficients. To understand the molecular mechanisms using the correlation-based integration method, selected DEGs in the time-series data and the correlation between the transcriptomic and metabolic changes in mice, were studied [90–92].

The clustering method using data set connection is one of the most conceptually simple methods for combining multiple omics data sets into a single model. It allows the grouping of omics data sets with similar properties, such as expression levels, for inferring basic connections and patterns. It includes hierarchical cluster analysis, K-means cluster analysis, and random forests approach [93,94]. These methods can distinguish between distinct and indistinct groups; however, it has limitations when the sizes of the connected data sets differ significantly and the pattern of elements in a small data set is dominated by the pattern of elements in the larger data set.

Multivariate analysis can integrate complex omics data sets, and is more powerfully applied in experimental design and metadata analysis. The most common multivariate techniques are principal component analysis and partial least squares [95]. This approach allows users to predict various aspects or trends of a data set, including the variance or covariance associations and to investigate dynamic relationships across transcripts, proteins, and metabolites [96].

### Function-based integration

When integrating various omics data, it is necessary to understand the data against the background of existing biological knowledge of how these molecules connect. Biological understanding can be improved through pathway mapping using previously identified databases [85–87,97]. The use of these databases to investigate changes and associations at the molecular level in response to specific environments and stimuli is well established for investigating the enriched pathways and expressed molecular mechanisms. The databases used as references for biological metabolic pathways are KEGG (https://www.genome.jp/kegg/), GO (http://geneontology.org/), and Reactome (https://reactome.org/). These databases can be applied to MOI analysis of the key information and pathways of omics data. However, pathway annotations across different species often provide insignificant results. Based on the correlation, the strength of the relationship across expressed molecules can be evaluated through co-expression analysis [98]. This analysis suggests important clusters, modules, and hubs for biological insights related to specific pathways or regulatory molecules in a variety of biological studies. Besides co-expression network analysis, there are various biological network-based analysis for identifying organisms and cellular mechanisms. Biological networks represent complex connections across different types of molecular elements, such as genes, proteins, and metabolites. These networks help construct subnetworks that do not rely on predefined knowledge.

## DISCUSSION

The MOI approach is limited by the differences in data output, variability in data structures, noise between data production platforms, and various data analysis algorithms. These biases account for inaccurate phenotypic changes. Accurate and continuous verification of experimental design, data production, databases, and data analysis tools are required for the meaningful biological interpretation of multi-omics data. Despite these complex problems and processes, data validation through various MOI approaches will increase data availability and enable integrated analysis. Many of the currently published multi-omics studies provide a rationale for what has been known or commonly observed for a long time, and some provide new insights. Applications include personalized health and nutrition, through identifying candidate genes, drug targets, and biomarkers. Detecting true causal genes, regulatory networks, and pathways will enable improving animal health, well-being, and production. Studies in a larger population will greatly increase the usefulness of predicting phenotypes, based on genetic and epigenetic variations. These approaches can reduce the repetitive work by different groups and provide a better understanding of the complex quantitative properties and underlying biology.

## CONCLUSION

Genetic studies are conducted to improve productivity indicators such as meat quality, disease susceptibility, and litter size in livestock science. The phenotype is influenced by environmental factors that modulate genetic factors; therefore, improving these indicators only through genetic factors has limitations. The molecular mechanism underlying epigenetics, in response to these environmental factors is not clearly understood. Epigenetic changes in livestock influence the physiological and developmental processes, through regulating gene expression. DNA methylation, a common mechanism of epigenetics, plays an important role in phenotypic variations. This review summarizes the mechanism of genetic regulation by epigenetic variations, methods of profiling epigenetic changes, and strategies for integrating omics data to understand molecular mechanisms. A comprehensive understanding of the epigenetic changes and the identification of novel factors could be a breakthrough for better genetic improvement in livestock.

## Figures and Tables

**Table 1:** Overview of comparison between microarray and RNA-seq approaches

Items	Microarray [99]	RNA-seq [56]
Principle	Hybridization	High-throughput sequencing
Resolution	Several to 100 bps	Single base
Reference genome required	Only knowledge about the microarray	The species or closely related species
Different isoform	Limited	Yes
Discover new transcript	Limited	Yes
Non-coding RNA	Limited	Yes

**Table 2:** Summary and comparison of the characteristics of global DNA methylation methods

Attributes	Affinity enrichment-based	Restriction enzyme-based	Bisulfite conversion
Assays	MeDIP-seq [81], MBD-seq [70]	HELP-seq [64], MRE-seq [65]	WGBS [46], RRBS [67]
Resolution	Approximately 150 bp	Single base	Single base
Regions covered	Approximately 23 million CpGs	Approximately 2 million CpGs	>28 million CpGs (WGBS) approximately 2 million CpGs (85% of CpG islands and 60% of promoters; RRBS)
Advantages	Allows for rapid and specific assessment of the average methylation levels of large DNA regions, No mutation introduced, Cost-effective	High sensitivity with lower costs, Simple approach, Cost-effective	Evaluates methylation status of every CpG site
Limitations	Limited by the quality and specificity of the antibody or protein, Bias into hyper-methylated regions, Unpredictable absolute methylation level, No information on separate CpG dinucleotides	Restricted to restriction enzyme-digestion sites, Requires large amount, high purity, and integrity of DNA	High cost, High DNA input, DNA damage after bisulfite conversion

MeDIP-seq, methylated DNA immunoprecipitation and sequencing; MBD-seq, methylated-CpG-binding protein sequencing; HELP-seq, *Hpa*II tiny fragments Enrichment by Ligation-mediated PCR; MRE-seq, methylation-sensitive restriction enzyme; WGBS, whole-genome bisulfite sequencing; RRBS, reduced-representation bisulfite sequencing; CpGs, cytosine phosphate guanine.

**Table 3:** Summary of the major multi-omics integration approaches

Integration method	Analysis method	Characteristics	Elements	Reference
Statistical-based	Correlation	Simplicity and intuitiveness	Pearson, Spearman	[100]
	Clustering using data set connection	Distinguish clear and unique groups	Hierarchical, K-means, random forests	[101]
		Highly dependent on the size between data sets		
	Multivariate	Powerfully applied in a metadata analysis	PCA, PLS	[102]
		Predict various aspects or trends of a data set		
Function-based	Reference database	Complex connections between various types of molecular elements	KEGG, GO, Reactome	[103]
		Differences exist in different species		
	Networking	Provides critical clusters, modules, and hubs	GCN, WGCNA	[104]
		Complex connections between various types of molecular elements		

PCA, principal component analysis; PLS, partial least squares; KEGG, Kyoto encyclopedia of genes and genomes; GO, gene ontology; GCN, gene co-expression network; WGCNA, weighted gene co-expression network analysis.
